# In *Salmonella* Typhimurium, YiiD Modulates cAMP Levels in Lag Phase During Growth on Succinate

**DOI:** 10.1111/mmi.70074

**Published:** 2026-05-03

**Authors:** John A. Ciemniecki, Jessica L. Will, Jorge C. Escalante‐Semerena

**Affiliations:** ^1^ Department of Microbiology University of Georgia Athens Georgia USA

**Keywords:** cAMP, fatty acid biosynthesis, lag phase, *Salmonella*, succinate metabolism, yeast Gcn5‐related proteins, YiiD protein

## Abstract

In *Salmonella* Typhimurium ( *S.* Typhimurium), *yiiD* encodes a two‐domain protein, with one domain having *bona fide* malonyl‐ACP decarboxylase activity, and the other showing sequence similarity to Gcn5‐type acetyltransferases (GNATs). Recent work on this enzyme was motivated by the essentiality of its decarboxylase domain to initiation of fatty acid biosynthesis in a strain devoid of β‐ketoacyl‐[acyl‐carrier‐protein, ACP] synthase III (FabH) activity. A function for the putative GNAT domain has not been established. We find that a ∆*yiiD* strain has an unconventional, slow‐growth lag phenotype during growth on succinate that is only weakly dependent on YiiD decarboxylase activity, implicating the putative GNAT domain. The ∆*yiiD* mutation suppresses the effect of a ∆*rpoS* mutation that is known to shorten the lag phase during growth on succinate, suggesting the function of the YiiD protein may also be regulatory. Isolation of spontaneous suppressor mutations in a ∆*yiiD* strain revealed changes in the promoter of *cpdA*, the gene encoding cyclic‐AMP phosphodiesterase. Exogenous addition of cAMP to the medium fully abrogated the ∆*yiiD* phenotype, and intracellular cAMP measurements revealed that the ∆*yiiD* strain fails to accumulate cAMP during lag phase, with levels about half of those measured in the wildtype strain. During the lag phase, the ∆*yiiD* strain was also measured to have increased expression of the adenylate cyclase gene, *cyaA*, implying that the mechanism altering cAMP levels occurs posttranscriptionally. We conclude YiiD function is necessary for early cAMP accumulation during transitions into some non‐phosphotransferase system (non‐PTS) growth conditions. We suggest changing the name of the YiiD protein to LcmM (for lipid and carbon metabolism modulator) to encapsulate its roles in *S.* Typhimurium physiology.

## Graphical abstract

The YiiD enzyme, found by others to perform a redundant role in fatty acid biosynthesis, is found to be unexpectedly required for the early increase in cAMP in lag phase during growth on succinate. This intriguing finding suggests a mechanistic link may exist between fatty acid biosynthesis and catabolite repression.

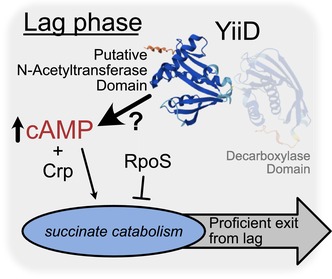

## 1 Introduction

**FIGURE 1:**
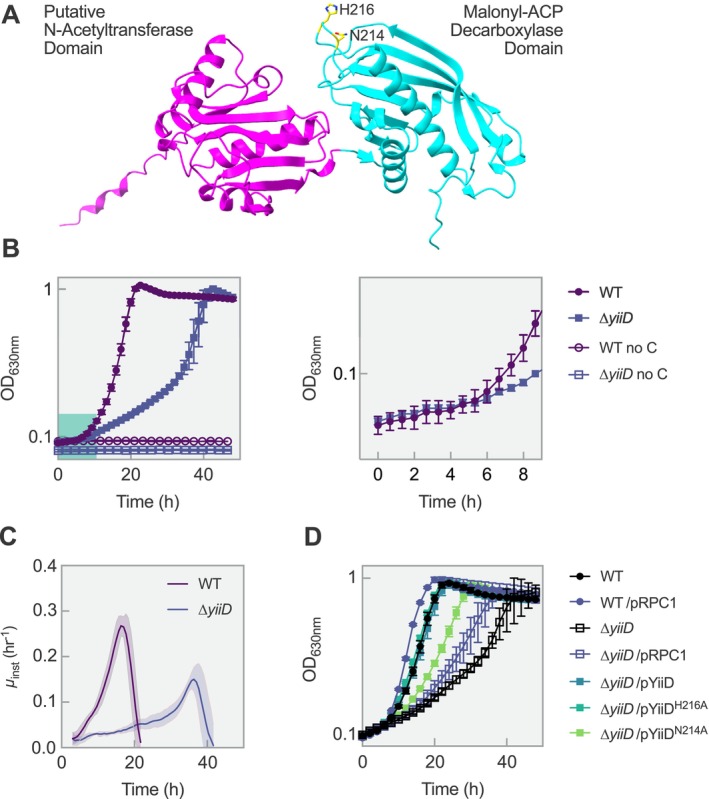
Loss of YiiD function causes a slow growth preceding the maximum growth rate on succinate. (A) AlphaFold DB model of YiiD (Identifier AF‐Q7CPC7‐F1) with domains colored. In yellow are key residues for decarboxylase activity, identified by Whaley et al. 2021. (B) Cultures grown in NCE minimal medium (see *Experimental procedures*) supplemented with or without succinate (30 mM). A zoomed‐in display of the 0–9 h period of early lag is presented in the right plot. (C) The instantaneous growth rates of A, plotted as a function of time, showing that the ∆*yiiD strain* displays a slow‐growth lag preceding transition to its maximum growth rate. Lines represent average of four biological replicates and shaded regions one standard deviation. (D) Complementation of ∆*yiiD in trans* during growth on succinate. The pRPC1 vector used places the *yiiD* ^*+*^ gene under a T7 promoter, but T7 polymerase was not necessary for the complementation. YiiD^H216A^ and YiiD^N214A^ variants have previously been found to possess 10‐ and 100‐fold decreases in decarboxylase activity, respectively. Growth curves are representative of at least three biological replicates. Error bars represent one standard deviation. In some cases, the error bars are smaller than the symbol, hence they are not shown.

YiiD is a two‐domain protein composed of an N‐terminal domain with putative Gcn5‐related N‐acetyltransferase (GNAT) activity, and a C‐terminal domain with *bona fide* malonyl‐ACP decarboxylase function (Figure 1A; Whaley et al. 2021). Prior work on the YiiD enzyme (also referred to as FabY in *Salmonella* Typhimurium or MadA in *Escherichia coli* ) identified it as essential in mutants lacking the ketoacyl‐ACP decarboxylase (*fabH*) enzyme that initiates fatty acid synthesis (FAS), with ∆*fabH ∆yiiD* mutants being synthetic lethal (Sanyal et al. 2019). It was found that the YiiD malonyl‐ACP decarboxylase activity allows for an alternative biosynthetic route to form the acetoacetyl‐ACP necessary for FAS initiation (Whaley et al. 2021). This alternative route consists of YiiD catalyzing the formation of acetyl‐ACP, which is used as a substrate with malonyl‐ACP to form the essential FAS precursor acetoacetyl‐ACP via the FabB or FabF 3‐ketoacyl synthases. Notably, YiiD is the only known enzyme to form acetyl‐ACP in the cell (Cronan 2023). The putative GNAT domain linked to the YiiD decarboxylase was dispensable for its function in FAS initiation and is not present in all homologs of the malonyl‐ACP decarboxylase across bacteria (Whaley et al. 2021), prompting the question of its role. Our group has a long‐standing interest in characterizing bacterial GNATs of unknown function and undertook this study to further probe the physiological role of YiiD.

We report here that *Salmonella* Typhimurium strains lacking YiiD function unexpectedly have a significant lag phenotype when grown on succinate as the sole source of carbon and energy. Succinate metabolism is of particular interest to *S.* Typhimurium researchers because it is a key metabolic signal that is necessary to upregulate pathogenicity islands during macrophage infection (Rosenberg et al. 2021). It is also a metabolite excreted by commensals in the gut, making it relevant to *S.* Typhimurium colonization (Schubert and Unden 2022). Despite its importance to such niches, cells display a relatively long lag during aerobic growth on succinate (Hersch et al. 2021). Recent work has found that multiple transcription factors contribute to the lag, including the cold‐shock protein C (CspC) RNA‐binding protein, the IscR Fe‐S cluster biosynthesis regulatory protein, the RbsR ribose operon repressor, and the RpoS sigma factor (Wenner et al. 2024). Intriguingly, mutations in any single one of these repression systems results in a near‐equivalent, large contraction of the lag time (Wenner et al. 2024), suggesting a floodgate effect where after a small threshold of repression is alleviated, all repression is effectively dispelled. However, the central repressor appears to be RpoS, as several of the repressors identified above exert their effect by altering the synthesis of RpoS (Wenner et al. 2024). RpoS represses expression of TCA cycle genes, and it has been correlated with decreased expression of *dctA*, encoding the aerobic C4‐dicarboxylate inner membrane transporter necessary to succinate uptake (Hersch et al. 2021). Interestingly, suppressor mutations mentioned above that do not have effects on RpoS levels in the cell (e.g., IscR) directly regulate the expression of *dctA* (Wenner et al. 2024). Altogether, these findings suggest a model where RpoS‐related suppressors are epistatic to *dctA*, and therefore the expression of the highly repressed *dctA* is what predominantly determines lag time on succinate.

Another major regulatory factor influencing *dctA* expression is the cAMP‐Crp and DcuR‐DcuS systems. Cyclic AMP (cAMP) formation occurs in response to a lack of PTS sugars in the medium and an elevated phosphoenolpyruvate‐to‐pyruvate ratio in the cell, which each elevate the phosphorylation state of EIIA^Glc^ that in turn activates the adenylate cyclase (Cya) enzyme that produces cAMP (Bettenbrock et al. 2007). Separately, DcuR is phosphorylated by DcuS in the presence of C4‐dicarboxylates, causing it to activate gene expression within its regulon, including *dctA* (Schubert and Unden 2022). Recent work in *Escherichia coli* has shown that cAMP‐Crp is required for both *dcuR* expression and DcuR activation of *dctA* expression, constructing a coherent feed‐forward loop (Schubert and Unden 2024). The net result of this regulatory topology is that sufficient cAMP‐Crp is required for transcription of *dctA* to occur, regardless of the DcuR phosphorylation state (Schubert and Unden 2024).

In this study, we demonstrate that the accumulation of cAMP during transition to growth on succinate is unexpectedly dependent upon the YiiD enzyme in a *fabH* ^*+*^ background. The mutant's failure to accumulate cAMP results in an unusual slow‐growth lag phenotype that is eventually overcome with time to achieve a fast growth rate with wildtype cAMP levels. Our results unveil a new physiological role for this enzyme in modulation of catabolite repression, suggesting it is mechanistically linked to fatty acid biosynthesis.

## 2 Results

### 2.1 YiiD Function Affects Growth on Succinate

**FIGURE 2:**
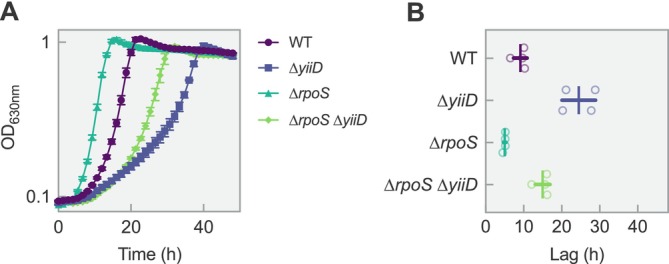
Loss of YiiD function suppresses a ∆*rpoS* mutation. (A) Growth analysis of the effect of ∆*yiiD* and ∆*rpoS* mutations on lag. Cultures grown as in Figure 1A. Plot is representative of four biological replicates. Error bars represent one standard deviation. (B) Lag times associated with growth of strains in A. Lag times are defined as the time elapsed before transition to the cultures' maximum growth rate, for calculation see *Experimental procedures*. Each open circle is a biological replicate; the vertical lines represent the mean, and the horizontal lines represent one standard deviation.

All strains generated and tested during this study were derived from a ∆*araB* strain to retain the ability to use arabinose‐inducible systems across derivative strains. We refer to ∆*araB* throughout the text as wildtype (WT in figures). We began investigating the physiological role of the YiiD enzyme by screening for growth phenotypes on TCA cycle intermediates, finding it had no discernable phenotype on citrate, isocitrate, malate, and fumarate. However, our search identified that a ∆*yiiD* strain displayed an extended lag time during succinate catabolism (Figure 1B). Plotting the instantaneous growth rate over the course of growth confirms that the ∆*yiiD* mutation causes an unusual slow growth during the lag phase preceding initiation of its maximum growth rate (Figure 1C). While the ∆*yiiD* strain also displayed a lower *μ*_max_ in this analysis, it was unclear if this was an artifact of partial nutrient depletion after the slow growth period. The slow growth was dependent on the presence of the supplied succinate and therefore not attributable to a diauxie of the spent medium carried over from the pre‐culture (Figure 1B). The lag time before transition to *μ*_max_ of the mutant was found, on average, to be 15 h longer than the lag time of the wildtype strain (Figure 2B). Expression of the *yiiD* ^+^ gene *in trans* corrected the phenotype of the mutant strain (Figure 1D). We also constructed variants of YiiD that have been previously characterized (Whaley et al. 2021) to diminish decarboxylase activity. A H216A variant (10‐fold decrease in decarboxylase activity) still complemented the ∆*yiiD* mutation, while a H214A variant (100‐fold decrease) partially complemented (Figure 1D). While possible effects of these mutations on the putative GNAT domain function are unknown, our results suggest the known decarboxylase function is largely dispensable. We concluded that the ∆*yiiD* strain has a non‐canonical lag phenotype where it is delayed in establishing its maximum growth rate on succinate but can still perform an initial slow growth. Loss of the N‐terminal putative GNAT domain appears more pertinent to the phenotype than the YiiD decarboxylase activity of the C‐terminal domain.

### 2.2 YiiD Function Opposes the Role of RpoS During Lag on Succinate

The unusually long lag during succinate catabolism in the pathogenic strain *Salmonella* Typhimurium 14028s is primarily caused by RpoS (Wenner et al. 2024). Expression of the *dctA* gene encoding the C4‐dicarboxylate transporter is upregulated in ∆*rpoS* strains, though it remains unclear if the repression is direct (Hersch et al. 2021). Accordingly, ∆*rpoS* strains have a much shorter lag during growth on succinate. While the *rpoS* gene in the *S.* Typhimurium LT2 strain we use in our study has a non‐canonical start codon that significantly lowers its expression (Wilmes‐Riesenberg et al. 1997), a ∆*rpoS* strain still displayed reduced lag on succinate (Figure 2A,B). Given our initial findings that YiiD affected lag, we wondered whether its function was dependent on the established RpoS regulatory role. To test this possibility, we generated null alleles of *rpoS* in both the wildtype and ∆*yiiD* strains. Intriguingly, the ∆*rpoS* ∆*yiiD* strain displayed an intermediate lag between wildtype and ∆*yiiD* (Figure 2A,B). Therefore, YiiD was necessary to the lag decrease of an ∆*rpoS* strain, and reciprocally, RpoS contributes to the extended lag present in a ∆*yiiD* strain. Altogether, these results suggest that YiiD may play a regulatory role within the succinate‐relevant regulon of RpoS but with an opposing, activating function.

### 2.3 The Succinate Phenotype of a Δ*yiiD* Strain Is Reversible by Mutations in Different Loci

As described in *Experimental procedures*, we obtained several independent, spontaneous derivatives of the Δ*yiiD* strain with succinate as the sole source of carbon and energy. The putative revertant strains were passaged selectively several times on NCE succinate agar to confirm reversion of the phenotype, then non‐selectively in liquid rich media before testing growth in liquid minimal medium alongside other strains. The genomes of the revertant strains were sequenced to identify the location and nature of the mutations, listed in Table 1.

**TABLE 1:** Mutations that decrease the lag of a Δ*yiiD* strain during growth with succinate.

Strain	WGS^a^ change	Genome location (nt)	Gene affected	# of reads	% Confidence^b^
JE26099	G→T; L28F	2,683,343	*iscR*	11,217,900	99.8
JE26100	Intergenic G→T; Type 2 IscR binding site	3,791,596	*dctA*	5,596,492	98.9
JE26102	Intergenic C→A (−7 bp from *cpdA* initiating ATG codon)	3,347,305	*cpdA*	3,013,718	97.2
JE26104	Intergenic C→A (−8 bp from *cpdA* initiating ATG codon)	3,347,306	*cpdA*	4,063,207	99.1

*Note:* *cpdA* encodes the 3′,5′‐cAMP phosphodiesterase; *iscR* encodes a member of the Rrf2 family of regulators (Nesbit et al. 2009) that represses its own expression and that of the iron–sulfur center biosynthetic operon (*iscSUA*); *dctA* encodes a C4‐dicarboxylate:proton symporter (Baker et al. 1996; Janausch et al. 2002).

^a^ WGS, whole genome sequencing.

^b^ Percentage of the total number of reads containing the reported mutation.

A recent study performed a comprehensive spontaneous mutant hunt and TnSeq to identify mutations in *S*. Typhimurium associated with decreased lag on succinate (Wenner et al. 2024). Two of the mutants we isolated in the ∆*yiiD* background corresponded to those found during that study in a *yiiD* ^*+*^ background: a point mutation in *iscR* (Fe‐S cluster regulator) and a mutation in the 5′‐UTR of *dctA* annotated as an IscR binding site (Wenner et al. 2024; Giel et al. 2006). Mutational analyses of these genes were consistent with the findings of that study, that is, deletion of *iscR* alleviates lag on succinate (Figure S1) and deletion of *dctA* abolishes growth (Figure S2).

However, we also isolated two mutants in the 5′‐UTR of *cpdA*, encoding cAMP phosphodiesterase. Importantly, this mutation was not isolated in the previously mentioned study and appears to be a suppressor specific to ∆*yiiD*. We therefore investigated these mutants further.

### 2.4 YiiD Function Modulates cAMP Levels During Lag Phase

**FIGURE 3:**
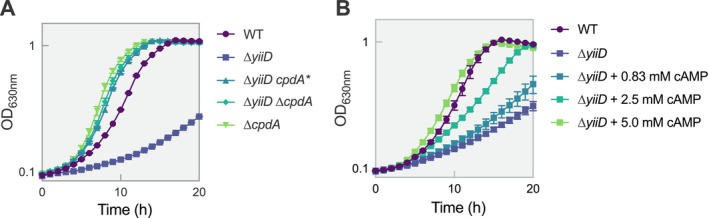
Loss of CpdA function suppresses a ∆*yiiD* mutation. Growth experiments were conducted in NCE supplemented with succinate (30 mM). (A) Growth of ∆*cpdA* strains and the spontaneous ∆yiiD cpdA* suppressor. (B) Dose‐dependent acceleration of the ∆*yiiD* strain's slow growth via cAMP supplementation. Growth curves are representative of at least three biological replicates. Error bars represent one standard deviation.

**FIGURE 4:**
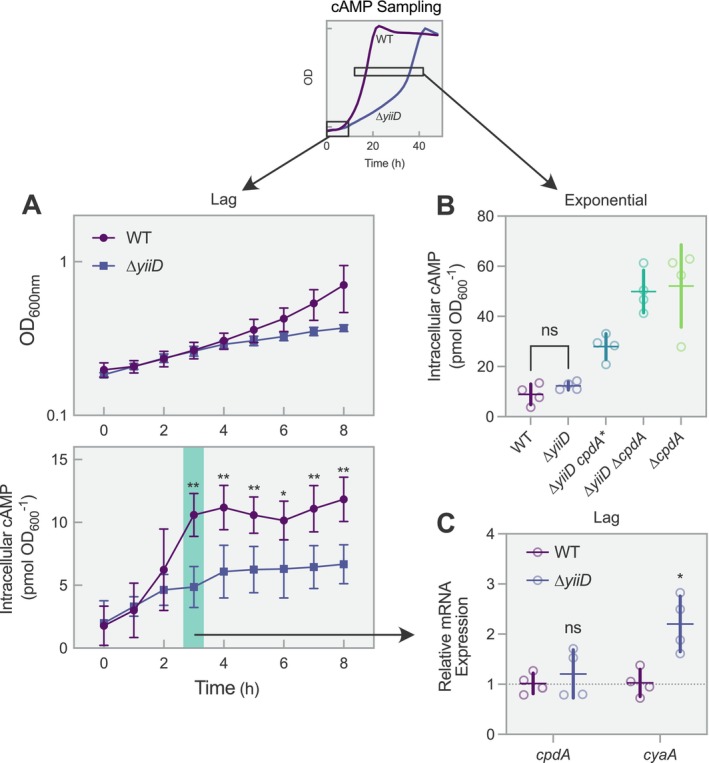
YiiD is required for early cAMP accumulation during adaptation to growth on succinate. (A) Measurement of intracellular cAMP accumulation during lag phase. Initial OD was increased in this experiment to obtain enough cell mass for measurement of early time points. Each point is the average of five biological replicates. (B) Intracellular cAMP levels during exponential phase. Samples were collected at different time points depending on the strain, but all at OD_600nm_ = 0.3. (C) RT‐qPCR measurement of mRNA encoding the two enzymes directly acting on cAMP in the cell, CpdA and Cya. Growth of cultures was conducted as in A with mRNA collected at the 3 h time point. In B and C, each open circle is a biological replicate; the horizontal bars are the mean. In all plots, vertical error bars represent one standard deviation, and asterisks indicate *p < 0.05 or **p < 0.01 using Welch's *t*‐test.

CpdA (3,5′‐cyclic‐AMP phosphodiesterase, EC 3.1.4.53) degrades cAMP, and accordingly deletion of *cpdA* increases cAMP levels in the cell (Inada et al. 1996; Figure 4B). Shown in Figure 3A is the strong positive effect upon the ∆*yiiD* phenotype of a one‐nucleotide change at position 7 upstream of the initiating codon of *cpdA*. A Δ*cpdA* Δ*yiiD* strain phenocopied the effect of the one‐nucleotide change (Figure 3A), implying that the spontaneous mutation that was isolated inhibits the transcription and/or translation of *cpdA*. This result prompted us to test the effect of exogenously added cAMP. Indeed, supplementation of the succinate medium with increasing amounts of cAMP had a dose‐dependent effect on the ∆*yiiD* mutant's initial slow growth period, fully complementing YiiD absence at 5.0 mM (Figure 3B).

Given that (1) various ways of increasing *dctA* expression (Wenner et al. 2024; Table 1) appear sufficient to reduce lag on succinate and (2) the cAMP‐Crp complex is an activator of *dctA* expression (Schubert and Unden 2024), our results could be interpreted as either an indirect or direct effect. That is, artificially increasing cAMP levels may indirectly overcome whatever other deficit is imposed by ∆*yiiD*, or a ∆*yiiD* strain is directly deficient in cAMP accumulation during lag phase. To discern between these scenarios, we measured intracellular cAMP levels via a cAMP ELISA assay using extracts collected from lag‐phase cells grown in succinate (Figure 4A). To obtain enough cell mass for accurate measurements during these early time points, we conducted our assay with an initial inoculum density of OD_600nm_ ~ 0.2, twice that of our previous growth experiments. We observed that over 1–3 h post inoculation, while the OD was still identical between the cultures, cAMP concentration rapidly increased ~7× in the wildtype strain but failed to do so in the ∆*yiiD* strain, establishing a ~2‐fold difference maintained between the cultures during lag (Figure 4A). In contrast, if the cultures were inoculated at a lower initial OD and measured at different timepoints but during their respective exponential phases (OD_600nm_ = 0.3), there was no significant difference in intracellular cAMP between the wildtype strain and the ∆*yiiD* strain (Figure 4B). To test whether the cAMP effect exerted by YiiD during lag mechanistically occurs at the transcriptional level, we measured the relative expression of *cpdA* and *cyaA* mRNA transcripts using RT‐qPCR at the 3 h time point when differences in intracellular cAMP are maximal. We found that loss of YiiD had no significant effect on *cpdA* expression but increased *cyaA* expression in the cell (Figure 4C), presumably in response to the lower intracellular levels of cAMP. This finding implies the mechanism connecting YiiD function to cAMP levels occurs posttranscriptionally. Overall, we concluded that YiiD loss causes a reduction of cAMP levels specifically during lag phase (Figure 4A,B). Given the initial slow growth rate could be tuned back to wildtype levels when cAMP was recouped via supplementation (Figure 3B), the effects of cAMP upon growth were sufficient to explain our observed phenotypes. Our findings show that YiiD is required for the early elevation of cAMP levels during lag on succinate.

## 3 Discussion

### 3.1 Expanding the Physiological Context of the YiiD Enzyme

Previous work on the putative bifunctional YiiD enzyme found that the C‐terminal domain of the protein has malonyl‐ACP decarboxylase activity that serves as the second known route toward generating the acetoacetyl‐ACP necessary for fatty acid synthesis initiation (Whaley et al. 2021). In this study, we have identified another role for this enzyme in modulating intracellular cAMP levels during lag phase (Figure 4A) that is predominantly dependent on some function independent of its decarboxylase activity (Figure 1C) and occurs posttranscriptionally (Figure 4C), potentially via its N‐terminal putative GNAT domain. We show that YiiD decreases lag on succinate via this cAMP function (Figures 1B and 3B). The link to cAMP is in line with our finding that the absence of YiiD opposes the known effects of the absence of RpoS on lag (Figure 2), as these global regulators have opposing effects on the expression of succinate catabolism genes (Hersch et al. 2021; Schubert and Unden 2024).

### 3.2 The YiiD Enzyme Fine Tunes cAMP Levels

That the ∆*yiiD* strain only displays a phenotype during growth on succinate is somewhat surprising because lowered cAMP levels could be expected to affect lag on more carbon sources. This discrepancy is likely reconciled by two points: first, the effect size of the cAMP concentration change is relatively small (~2× vs. the ~10×) increase measurable upon glucose depletion (Makman and Sutherland 1965, Notley‐McRobb et al. 1997), meaning that YiiD likely functions to tune cAMP levels as opposed to acting as a master controller like the PTS system (Bettenbrock et al. 2007; Epstein et al. 1975). Second, in *S.* Typhimurium, *dctA* repression is strong with a concomitant lag on succinate that is unusually long (Hersch et al. 2021). It therefore seems likely that the ∆*yiiD* phenotype was detectable because this anomaly unique to *S.* Typhimurium afforded a growth measure with unusual sensitivity to cAMP levels. This is also likely why there is not a similar detectable phenotype in *E. coli* (Figure S3), despite their respective YiiD enzymes' 94% amino acid sequence identity conservation. Future studies may therefore be able to leverage lag on succinate in *S.* Typhimurium to sensitively assess other factors that putatively fine‐tune cAMP levels in the cell.

### 3.3 Possible Ways That the YiiD Enzyme Benefits Cells and Alters cAMP Levels

Our interpretation of the data reported herein prompts the question of what the adaptive advantage of such a fine‐tuning mechanism could be. We note that the decarboxylase domain of the YiiD enzyme is the only known producer of acetyl‐ACP in the cell, and its putative GNAT domain may acetylate an undetermined target using acetyl‐CoA. We also note the expression of *yiiD* is significantly increased during the stringent response (Sanyal et al. 2019), implying that its role is predominantly relevant during periods of C/N limitation or transition. While sugar uptake (Peterkofsky and Gazdar 1974) and the phosphoenolpyruvate‐to‐pyruvate ratio (Hogema et al. 1998) are the predominant signal inputs affecting the EIIA^Glc^ phosphorylation state, Cya activity, and cAMP levels, we speculate that during nutrient transitions YiiD links acetyl‐CoA and/or acetyl‐ACP levels to cAMP levels in the cell. Increasing concentrations of either acylated carrier would indicate the presence of a carbon source and therefore correlate with an advantageous time to moderately upregulate the cAMP‐Crp regulon above basal levels. Such a function would be adaptive during transitions from nutrient limitation to a growth condition with a non‐PTS carbon source. Indeed, a similar function for acetyl‐CoA has been speculated to exist for the cAMP‐Crp system before (Makman and Sutherland 1965) inspired by the fact that acetyl‐CoA is a central, carbon‐status‐integrative metabolite. However, this idea assumes the mechanistic link connecting YiiD to cAMP modulation is directly or closely involved in cAMP production or degradation, for example, Cya, CpdA, Crp, the phosphoenolpyruvate‐to‐pyruvate ratio, or components of the PTS system. Identifying the biochemical function of the putative GNAT domain, and its potential target, is therefore necessary to validate this physiological role.

### 3.4 Concluding Remarks

Commentary has been published about the desire for a more holistic understanding of cAMP dynamics (Green et al. 2014; Narang 2009) that resolves observations unexplained by the PTS‐dependent control of Cya activity (Inada et al. 1996; Notley‐McRobb et al. 1997). While it is unlikely YiiD alone could explain these discrepancies, here we show an intriguing example of PTS‐sugar‐independent cAMP modulation that potentially links it to fatty acid biosynthesis, and by extension, carbon status. The mutational/functional analysis of YiiD variants may therefore represent a tool of interest to help understand these finer points of cAMP regulation in the cell. Given the established function of the C‐terminal domain of YiiD in fatty acid metabolism and the involvement of the N‐terminal domain in the modulation of cAMP biosynthesis, we suggest changing the name of the YiiD protein to LcmM (for lipid and carbon metabolism modulator) to encapsulate its roles in *S.* Typhimurium physiology.

## 4 Experimental Procedures

### 4.1 Strain Construction and Growth Conditions

Studies were conducted with *Salmonella enterica* subsp. *enterica* sv. Typhimurium str. LT2. All strains (Table S1) were derived from a ∆*araB9* strain (referred to as wildtype in this study) unable to catabolize arabinose. Mutants were constructed using standard recombination procedures (Datsenko and Wanner 2000), reconstructed via bacteriophage P22 transduction (Davis et al. 1980), then resolved leaving in‐frame deletions of the genes. Deletions were confirmed via PCR and sequencing. Plasmids were constructed using BspQ1 restriction cloning (VanDrisse and Escalante‐Semerena 2016) and strains were transformed using electroporation. For experiments, strains were streaked out from DMSO stocks stored at −80°C onto LB plates and after growth were stored at 4°C for no longer than a week. All media were prepared with deionized water containing less than 10 ppb total organic carbon as measured by a Millipore Milli‐Q IQ A10 water monitor. In all cases unless otherwise noted, liquid cultures were incubated in slanted culture tubes at 37°C, 180 RPM. Overnight NB broth precultures (16–20 h, final OD_600nM_ ~ 2) were used to inoculate a minimal medium for growth experiments with a 2% (v/v) inoculum. The minimal medium used was No Carbon E medium (NCE medium, Berkowitz et al. 1968) consisting of K_2_HPO_4_ (28.3 mM), KH_2_PO_4_ (29.0 mM), NaNH_4_HPO_4_ (16.7 mM), MgSO_4_ (1 mM) and trace metals (Atlas 1995). All growth curves were measured using a Biotek ELx808 instrument set to 37°C with medium shaking. The 96‐well plate edges were wrapped twice in parafilm to minimize evaporation from the edge wells. For the cAMP measurements, the precultures were pelleted (6000 *g* for 3 min) three times with resuspension in NCE medium before inoculation into 50 mL of the growth medium supplemented with 0.005% (w/v) casamino acids in 250‐mL flasks.

### 4.2 Quantification of Growth Rate and Lag

We define lag as the amount of time elapsed before initiation of the cultures' maximum growth rate. To calculate growth rate and lag, we used a rolling‐window algorithm that scanned through the semi‐log growth curve data using a 3‐h window size to calculate the instantaneous growth rate, *μ*_inst_ as a function of time. The X‐intercept of the best‐fit line corresponding to the maximum growth rate is reported as the lag time. We note that for clarity, all growth curves are displayed with three out of every four consecutive points omitted, but this analysis was conducted with the full datasets. The analysis was done in Python 3.4.

### 4.3 Isolation of Spontaneous Suppressors

Cultures of the ∆*yiiD* strain were initiated in NB liquid and grown overnight at 37°C with shaking at 180 rpm. The cultures were washed in saline (NaCl, 0.85% w/v) and serially diluted. Dilutions were plated on 1.5% (w/v) noble agar containing NCE minimal medium with succinate (30 mM). Growth was monitored by imaging plates every 8 h for 3 d. Large, early‐arising colonies were selected and passaged three times on the same medium before non‐selective growth in liquid LB and storage. Verification of phenotype was performed as described above in a BioTek reader with succinate (30 mM) as sole carbon and energy source. Illumina Whole Genome Sequencing was performed using SeqCenter (Pittsburgh, PA).

### 4.4 cAMP Measurements

cAMP was measured using an ELISA kit (Cat. 581001; Cayman Chemical) following manufacturer instructions under the non‐acetylated cAMP protocol. A volume of culture equal to 0.375 OD units was periodically sampled from 50‐mL cultures and pelleted at 9000 *g* for 3 min. cAMP was extracted from the pellet via resuspension in 250 μL of HCl (0.1 N) and incubation for 20 min at RT. The suspension was then pelleted at 1000 *g* for 10 min, and the supernatant stored at −80°C until measurement. The samples were diluted by half in the manufacturer's ELISA Buffer to neutralize the acid prior to plate addition.

### 4.5 RNA Isolation and RT‐qPCR

Culture samples (25 mL) were centrifuged (6000 *g* for 2 min), the supernatants discarded, the pellets flash‐frozen in liquid N_2_, and then stored at −80°C before processing. RNA was isolated following the RNAsnap protocol (Stead et al. 2012). Subsequent RNase‐free DNase I treatment was conducted using the Ambion Turbo DNA‐free kit according to the manufacturer's protocol (Thermo Fisher Scientific). A small aliquot of each sample was measured for quality analysis using the RNA IQ protocol and Qbit fluorimeter (Invitrogen, Thermo Fisher Scientific). RNA (620 ng) from each sample was used for the synthesis of cDNA using the iScript cDNA synthesis kit according to the manufacturer's protocol (Bio‐Rad Laboratories). Primers for qPCR were designed using primer 3 software and were evaluated for specificity and melting curve prior to running the qPCR. For real‐time PCR, 20 μL reaction mixtures were prepared with 10 μL of 2× FastSYBR green master mix (Applied Biosystems), 500 nM each gene‐specific primer, and 15 ng of the cDNA. Each biological replicate was measured in technical quadruplet. The real‐time PCR was performed using a 7500 Fast real‐time PCR system (Applied Biosystems). Cycle threshold (*C*_T_) data were normalized to the *gyrB* gene. These normalized values (∆*C*_T_) were then normalized again against the average ∆*C*_T_ values of WT per gene to obtain ∆∆*C*_T_ values. Reported relative expression levels were calculated as $2^{-\Delta \Delta C_{T}}$.

### 4.6 Plotting and Statistical Analysis

All plotting and *t*‐tests were conducted in GraphPad Prism version 10.6.

## Author Contributions

**Jessica L. Will:** conceptualization, investigation, validation, formal analysis, data curation, visualization, writing – review and editing. **Jorge C. Escalante‐Semerena:** conceptualization, data curation, supervision, formal analysis, validation, investigation, funding acquisition, visualization, project administration, resources, writing – original draft, writing – review and editing. **John A. Ciemniecki:** conceptualization, investigation, validation, formal analysis, data curation, visualization, writing – original draft, writing – review and editing.

## Funding

This work was supported by the National Institutes of Health, R35GM130399. Life Sciences Research Foundation.

## Ethics Statement

The authors have nothing to report.

## Conflicts of Interest

The authors declare no conflicts of interest.

## Supporting information


**Table S1:** Strains and plasmids used in this work.
**Figure S1:** A single‐amino acid change in IscR or the absence of IscR circumvents the need for YiiD function during growth with succinate as the sole source of carbon and energy. Growth was measured in NCE medium supplemented with succinate (30 mM). The experiment is representative of biological duplicates. The asterisk represents a G‐to‐T mutation at nucleotide # 83 of the *iscR* gene, the resulting allele encoded variant IscR^L28F^.
**Figure S2:** A single nucleotide change in the dctA regulatory region circumvents the need for YiiD function with succinate as the sole source of carbon and energy. Growth was measured in NCE medium supplemented with succinate (30 mM). The experiment is representative of biological duplicates. The asterisk represents a G‐to‐T mutation at nucleotide #3,791,596 located within the *dctA* regulatory region.
**Figure S3:** Loss of YiiD function does not affect growth of *Escherichia coli* MG1655 on succinate. Growth analysis of the effect of ∆*yiiD* on NCE minimal medium supplemented with 30 mM succinate. Cultures grown as in Figure 1A. Plot is representative of five biological replicates. Error bars represent one standard deviation.

## Data Availability

All data generated during this work are reported in this paper and its Supporting Information file.
