# Supplementary material for: In Salmonella Typhimurium, YiiD Modulates cAMP Levels in Lag Phase During Growth on Succinate
Source: Mol Microbiol. 2026 May 3;126(1):15–23. doi: 10.1111/mmi.70074 (PMC13353551; doi:10.1111/mmi.70074)
Supplement: Supplementary file 1 — Table S1: Strains and plasmids used in this work. Figure S1: A single‐amino acid change in IscR or the absence of IscR circumvents the need for YiiD function during growth with succinate as the sole source of carbon and energy. Growth was measured in NCE medium supplemented with succinate (30 mM). The experiment is representative of biological duplicates. The asterisk represents a G‐to‐T mutation at nucleotide # 83 of the iscR gene, the resulting allele encoded variant IscRL28F. Figure S2: A single nucleotide change in the dctA regulatory region circumvents the need for YiiD function with succinate as the sole source of carbon and energy. Growth was measured in NCE medium supplemented with succinate (30 mM). The experiment is representative of biological duplicates. The asterisk represents a G‐to‐T mutation at nucleotide #3,791,596 located within the dctA regulatory region. Figure S3: Loss of YiiD function does not affect growth of Escherichia coli MG1655 on succinate. Growth analysis of the effect of ∆yiiD on NCE minimal medium supplemented with 30 mM succinate. Cultures grown as in Figure 1A. Plot is representative of five biological replicates. Error bars represent one standard deviation. [file MMI-126-15-s001.pdf]

SUPPLEMENTARY MATERIAL

In *Salmonella*, YiiD modulates cAMP in lag phase during growth on succinate.

John A. Ciemniecki[§], Jessica L. Will[§], and Jorge C. Escalante-Semerena*
Department of Microbiology, University of Georgia, Athens

ORCID
JAC: 0000-0003-2789-6700
JLW: 0000-0002-3229-3667
JCES: 0000-0001-7428-2811

[§]These authors contributed equally to this work

*Corresponding author: Department of Microbiology, University of Georgia, 330G Cedar Street Building C, 140 Cedar St, Athens, GA 30602 USA, T: +1 (706)-542-2651, E-mail: jcescala@uga.edu

Table S1. Strains and plasmids used in this work.

S. **Typhimurium[a] strains**	**Genotype**	**Reference/source[b]**
JE10079 (WT)	Δ*araB9*	Laboratory collection
**Derivatives of JE10079**		
JE23531	Δ*yiiD402*	
JE27639	WT /pRPC1 *kan*⁺	
JE26417	WT /pYiiD28 *kan*⁺ *yiiD*⁺	
JE27647	Δ*yiiD402* /pRPC1	
JE26421	Δ*yiiD402* /pYiiD28	
JE28422	Δ*rpoS1117*	
JE28515	Δ*rpoS1117* Δ*yiiD402*	
JE26099	Δ*yiiD402 iscR103* (encodes IscR^{L28F})	
JE10433	Δ*iscR101*	(1)
JE26943	Δ*yiiD402* Δ*iscR101*	
JE26100	Δ*yiiD402* G-to-T change @ nt# 3,791,596 (within the Type 2 IscR-binding site controlling *dctA* expression)	
JE25676	Δ*dctA81*	
JE25677	Δ*yiiD402* Δ*dctA81*	
JE26102	Δ*yiiD402* C-to-A change @ nt# 3,347,305 (-7 bp from *cpdA* initiating ATG codon)	
JE26104	Δ*yiiD402* C-to-A change @ nt# 3,347,306 (-8 bp from *cpdA* initiating ATG codon)	
JE28750	Δ*cpdA552*	(2)
JE28751	Δ*yiiD402* Δ*cpdA552*	
***Escherichia coli* strains**		
JE11631	MG1655 Δ*araBAD* Δ*araC*	Laboratory collection
JE11835	Δ*araBAD* Δ*araC yiiD750*::*kan*⁺	Laboratory collection
JE26778	Δ*araBAD* Δ*araC* Δ*yiiD751*	

[a]*Salmonella enterica* subsp. *enterica* sv. Typhimurium str. LT2
[b]Unless otherwise indicated, strains were constructed during this work

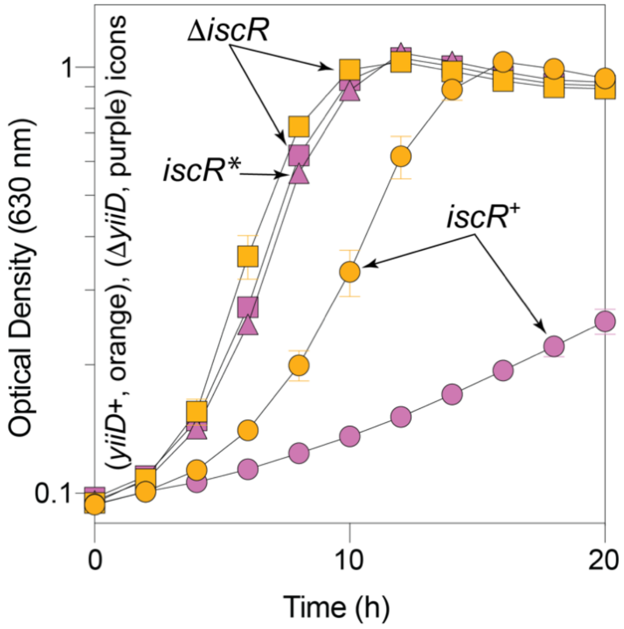

Figure S1. A single-amino acid change in IscR or the absence of IscR circumvents the need for YiiD function during growth with succinate as the sole source of carbon and energy. Growth was measured in NCE medium supplemented with succinate (30 mM). The experiment is representative of biological duplicates. The asterisk represents a G-to-T mutation at nucleotide # 83 of the *iscR* gene, the resulting allele encoded variant $IscR^{L28F}$.

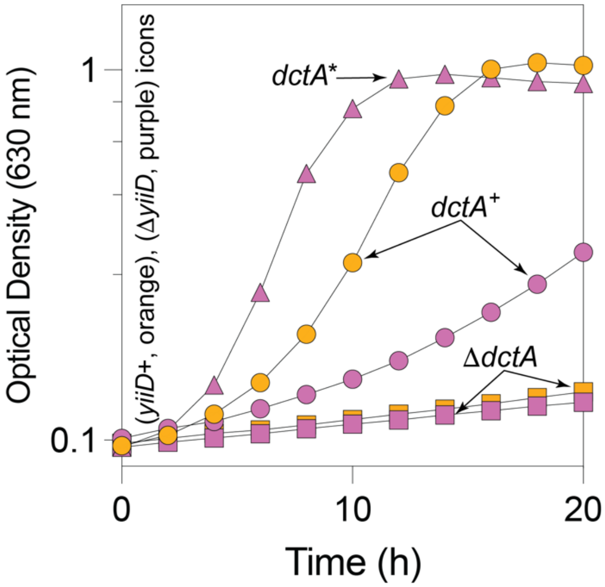

Figure S2. A single nucleotide change in the *dctA* regulatory region circumvents the need for YiiD function with succinate as the sole source of carbon and energy. Growth was measured in NCE medium supplemented with succinate (30 mM). The experiment is representative of biological duplicates. The asterisk represents a G-to-T mutation at nucleotide #3,791,596 located within the *dctA* regulatory region.

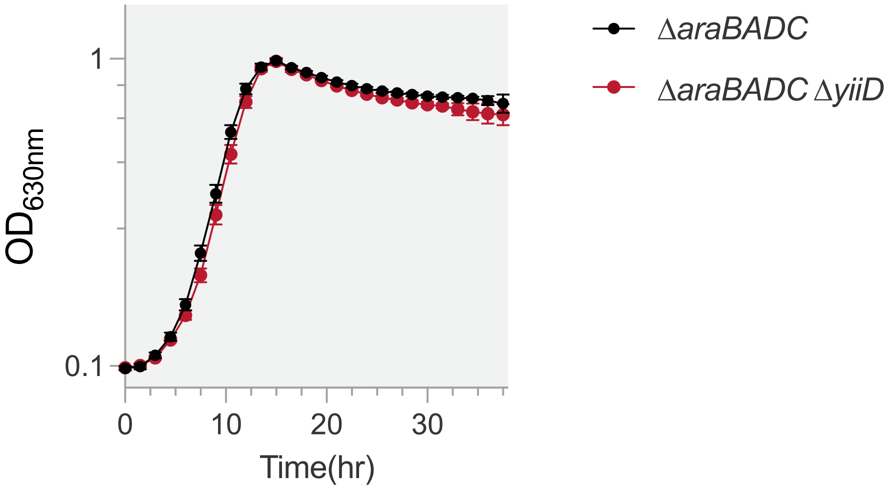

Figure S3. Loss of YiiD function does not affect growth of *Escherichia coli* MG1655 on succinate. Growth analysis of the effect of Δ*yiiD* on NCE minimal medium supplemented with 30 mM succinate. Cultures grown as in Figure 1A. Plot is representative of five biological replicates. Error bars represent one standard deviation.

References

1. Lewis JA, Boyd JM, Downs DM, Escalante-Semerena JC. 2009. Involvement of the Cra global regulatory protein in the expression of the *iscRSUA* operon, revealed during studies of tricarballylate catabolism in *Salmonella enterica*. J Bacteriol 191:2069-2076.
2. Bazurto JV, Dearth SP, Tague ED, Campagna SR, Downs DM. 2017. Untargeted metabolomics confirms and extends the understanding of the impact of aminoimidazole carboxamide ribotide (AICAR) in the metabolic network of *Salmonella enterica*. Microb Cell 5:74-87.
